# Two‐Year Follow‐Up of Patients With Atrial Fibrillation Receiving Edoxaban in Routine Clinical Practice: Results From the Global ETNA‐AF Program

**DOI:** 10.1002/clc.70091

**Published:** 2025-02-27

**Authors:** Raffaele De Caterina, Martin Unverdorben, Cathy Chen, Eue‐Keun Choi, Yukihiro Koretsune, Doralisa Morrone, Ladislav Pecen, Peter Bramlage, Chun‐Chieh Wang, Takeshi Yamashita, Paulus Kirchhof

**Affiliations:** ^1^ Chair of Cardiology University of Pisa and Cardiology Division, Pisa University Hospital Pisa Italy; ^2^ Fondazione Villa Serena per la Ricerca Città Sant'Angelo Italy; ^3^ Daiichi Sankyo, Inc. Basking Ridge USA; ^4^ Department of Internal Medicine Seoul National University College of Medicine and Seoul National University Hospital Seoul Republic of Korea; ^5^ National Hospital Organization Osaka National Hospital Osaka Japan; ^6^ Department of Surgical Division of Cardiology, Medical and Molecular Pathology and Critical Care Medicine University of Pisa Pisa Italy; ^7^ Institute of Computer Science of the Czech Academy of Sciences Prague Czech Republic; ^8^ Medical Faculty Charles University Pilsen Czech Republic; ^9^ Institute for Pharmacology and Preventive Medicine Cloppenburg Germany; ^10^ Department of Cardiology, Chang Gung Memorial Hospital Chang Gung University Taoyuan Taiwan; ^11^ Department of Cardiovascular Medicine Cardiovascular Institute Tokyo Japan; ^12^ Center for Cardiovascular Research University of Birmingham and SWBH and UHB NHS Trusts Birmingham UK; ^13^ Department of Cardiology University Heart and Vascular Center Hamburg Hamburg Germany; ^14^ German Center for Cardiovascular Research (DZHK) Partner Site Hamburg/Kiel/Lübeck Lübeck Germany

**Keywords:** anticoagulation, atrial fibrillation, direct oral anticoagulant (DOAC), edoxaban, major bleeding, oral anticoagulants, stroke prevention

## Abstract

**Background:**

Randomized clinical trials demonstrated similar efficacy and improved safety of direct oral anticoagulants versus warfarin in patients with atrial fibrillation (AF). Long‐term data in routine clinical practice are needed.

**Hypothesis:**

Patients with AF receiving edoxaban at baseline continue to have low annualized effectiveness and safety event rates in the second year of follow‐up, with regional variations observed.

**Methods:**

The Global ETNA‐AF program is a prospective, noninterventional study of patients with AF receiving edoxaban. Patient characteristics and annualized clinical event rates were assessed overall and by region across the 2‐year follow‐up. Annualized event rates of bleeding and thromboembolic events were assessed within the first year and conditionally in patients who were event‐free up to 12 months in the second year.

**Results:**

This analysis comprised 26 805 patients from Europe (*n* = 13 164), Japan (*n* = 10 342), and non‐Japanese Asian regions (*n* = 3299). Patients from Europe had the highest burden of comorbidities. The annualized event rates for major bleeding, any stroke, all‐cause death, and cardiovascular death varied by region. The global annualized event rates in the first and second year were 1.31%/year and 0.86%/year for major bleeding, 1.06%/year and 0.65%/year for any stroke, 0.84%/year and 0.73%/year for cardiovascular death, and 3.05%/year and 3.18%/year for all‐cause death.

**Conclusion:**

Annualized event rates for any stroke and major bleeding remained low through 2‐year follow‐up for patients with AF receiving edoxaban at baseline. Differences in annualized event rates for all‐cause and cardiovascular mortality between Europe, Japan, and non‐Japanese Asian regions may reflect variations in baseline characteristics.

**Trial Registration:**

Europe, NCT02944019; Japan, UMIN000017011; Korea/Taiwan, NCT02951039; Hong Kong, NCT03247582; and Thailand, NCT03247569.

## Graphical abstract

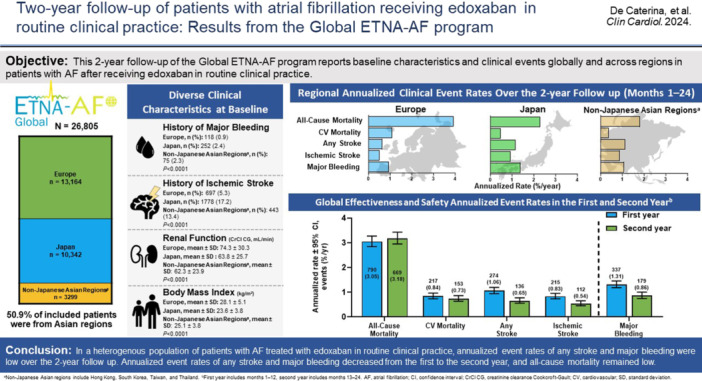

**Abbreviations**
AF: atrial fibrillation
BMI: body mass index
CHA_2_DS_2_‐VASc: Congestive heart failure, Hypertension, Age ≥ 75 years (doubled), Diabetes mellitus, Stroke (doubled), Vascular disease, Age 65–74 years, Sex category (female)
CI: confidence interval
CIF: cumulative incidence function
COPD: chronic obstructive pulmonary disease
CrCl: creatinine clearance
CrCl CG: creatinine clearance Cockcroft‐Gault
CRNM: clinically relevant nonmajor
CV: cardiovascular
DOAC: direct oral anticoagulant
ETNA: Edoxaban Treatment in routiNe clinical prActice
GI: gastrointestinal
HAS‐BLED: Hypertension, Abnormal renal/liver function, Stroke, Bleeding history or predisposition, Labile international normalized ratio, Elderly, Drugs/alcohol concomitantly
HF: heart failure
IQR: interquartile range
ISTH: International Society on Thrombosis and Haemostasis
MI: myocardial infarction
NA: not applicable
SD: standard deviation
SEE: systemic embolic event
TIA: transient ischemic attack
VKA: vitamin K antagonist

## 1 Introduction

The annual risk of any stroke for patients with atrial fibrillation (AF) is between 1% and 20% [1]. Direct oral anticoagulants (DOACs), such as edoxaban, are preferred over vitamin K antagonists (VKAs) in patients with AF for the prevention of ischemic stroke [2, 3]. In a meta‐analysis of four pivotal studies, DOACs were associated with a combined 19% risk reduction for any stroke and systemic embolic events (SEEs), a 51% reduction in hemorrhagic stroke, and a 10% risk reduction of all‐cause mortality compared with VKAs [3]. Major society guidelines recommend DOACs as the preferred drug class for stroke prevention in eligible patients with AF [4, 5, 6]. In the ENGAGE AF‐TIMI 48 trial, treatment with edoxaban versus warfarin prevented any stroke or SEEs with similar efficacy, lower rates of bleeding, and significantly lower rates of death from cardiovascular (CV) causes [2].

Randomized controlled trials have strict inclusion criteria, which limit the selection of patients [7, 8, 9]. In addition, randomized controlled trials have historically included limited representation from various geographical regions, including Asian countries. These limitations have contributed to trial populations that may not fully capture the diversity observed in real‐world settings [9, 10, 11]. Noninterventional studies overcome this limitation by including patients without specific criteria or restrictions, leading to a more diverse study population. Data from real‐world and randomized controlled trials are consistent and show that dabigatran [12], rivaroxaban [13, 14], apixaban [15], and edoxaban [8], effectively prevent thromboembolic events and exhibit similar or decreased risk of major bleeding compared with VKAs [3]. Given these findings, additional data from real‐world analyses are needed demonstrating the effectiveness and safety of edoxaban in a diverse patient population over a long‐term follow‐up.

The Global Edoxaban Treatment in routiNe clinical prActice (ETNA)‐AF is a prospective, observational noninterventional program evaluating the effectiveness and safety of edoxaban in patients with AF [7, 8]. The Global ETNA‐AF program is the largest prospective, observational program of a single DOAC to date, consisting of approximately equal numbers of patients from Asia and Europe; therefore, it provides the unique opportunity to assess the effectiveness and safety of edoxaban in different regions and ethnicities. At the 1‐year follow‐up, patients with AF receiving edoxaban at baseline had a low incidence of any stroke (ischemic stroke and hemorrhagic stroke), intracranial hemorrhage, and other major bleeding events in routine care [8].

This 2‐year follow‐up of the Global ETNA‐AF program reports baseline characteristics and clinical events globally and across regions in patients with AF receiving edoxaban in routine clinical practice.

## 2 Materials and Methods

### 2.1 Study Design

The study design of the ETNA‐AF program was published previously [7]. The ETNA‐AF program integrates data from several prospective, observational, and noninterventional studies from Europe (Germany, Austria, Switzerland, Belgium, Italy, Spain, the United Kingdom, Ireland, the Netherlands, and Portugal), Japan, and non‐Japanese Asian regions (Hong Kong, South Korea, Taiwan, and Thailand) [7]. The studies were registered in Europe (NCT02944019), Japan (UMIN000017011), Hong Kong (NCT03247582), South Korea/Taiwan (NCT02951039), and Thailand (NCT03247569). Sites included hospitals and outpatient clinics. The responsible ethics committees and institutional review boards approved the study protocols, except in Japan, where such approval is not required for this study type. The program complied with the Declaration of Helsinki and the International Conference on Harmonisation of Good Clinical Practice standards.

### 2.2 Inclusion and Exclusion Criteria

For ETNA‐AF, eligible patients included were those treated with edoxaban for AF according to the local label. In Japan, patients were included only if they were receiving edoxaban for the first time to prevent ischemic stroke and SEEs. All ETNA‐AF participants provided written consent before enrollment; patients who were part of a simultaneous interventional study were excluded from participation.

### 2.3 Study Drug

According to the label, the prescribed edoxaban dose at baseline was 60 mg once daily in patients who did not have renal impairment (creatinine clearance, 15 to ≤ 50 mL/min), weight ≤ 60 kg, or concomitant use of certain P‐glycoprotein inhibitors. Due to the observational nature of the study, patients could stop edoxaban or switch to another treatment at any time.

### 2.4 Assessments and Event Rates

Baseline information assessed included demographics, vital signs, history of AF and diagnosis, renal and hepatic parameters, bleeding history, and edoxaban treatment details. Medical history, clinical events, and descriptions had a code assigned from MedDRA (Medical Dictionary for Regulatory Activities). Effectiveness events evaluated in this analysis included all‐cause mortality, CV mortality, myocardial infarction (MI), any stroke, ischemic stroke, transient ischemic attack (TIA), hemorrhagic stroke, and SEEs. Safety events included major bleeding, intracranial hemorrhage, major gastrointestinal (GI) bleeding, clinically relevant nonmajor (CRNM) bleeding, and minor bleeding in accordance with the International Society on Thrombosis and Haemostasis definition [16]. Clinical events were reported based on physician diagnosis and assessment.

### 2.5 Statistical Analysis

Data presented are from the overall Global ETNA‐AF full analysis set, and events are included whether on or off edoxaban treatment. Results are presented using summary statistics (*n*, mean ± standard deviation [SD]; or median [interquartile range (IQR)]) for continuous variables and absolute and relative frequencies for categorical variables (*n*, %). *p* values for the testing of differences between the regions are based on a chi‐square test for categorical data and the Kruskal–Wallis test for continuous variables.

Overall and regional annualized clinical event rates were assessed across the 2‐year (months 1–24) follow‐up. Global and regional annualized clinical event rates were assessed in the first (months 1–12) and second year (months 13–24) based on time to the first event. As such, second year annualized event rates were calculated based on the first event that occurred during months 13–24 in patients who were event‐free from the first year. The number of patients with at least one clinical event is presented separately for each type of clinical event, and the time‐to‐event data are presented as annualized rates (cases per 100 patient‐years and displayed as %/year with a 95% confidence interval [CI]). As a sensitivity analysis, recurrent events were considered, allowing patients to contribute multiple events per year and at the same time contribute to both the first and second year annualized event rates, with time under observation within each year used as the person‐year denominator. Cumulative incidence function (CIF) of clinical events was performed unconditionally (1–24 months) and conditionally (13–24 months among event‐free patients) and calculated based on the formula 1 − S(t), where S(t) is the Kaplan–Meier estimate of the survival function. CIF curves were calculated for events between 1 and 24 months and, conditional on being event‐free at 12 months, from 13 to 24 months.

## 3 Results

### 3.1 Patient Population

In total, 26 805 patients were included in this 2‐year analysis of the Global ETNA‐AF program, with 13 164 (49.1%) from Europe, 10 342 (38.6%) from Japan, and 3299 (12.3%) from non‐Japanese Asian regions. During the 2‐year follow‐up, 3530 (13.2%) patients permanently discontinued edoxaban therapy, with higher proportions in Europe (16.1%) and non‐Japanese Asian regions (17.6%) than in Japan (8.0%).

### 3.2 Edoxaban Treatment

Overall, 13 918 (54.5%) patients received edoxaban 60 mg at baseline, with substantially higher proportions of patients in Europe (76.0%) than in non‐Japanese Asian regions (49.6%) and Japan (27.9%). In total, 68.3% of the patients received the recommended dose according to the local label (recommended 60 mg or recommended 30 mg), with 72.0% of patients in Europe, 64.1% of patients in Japan, and 65.9% of patients in non‐Japanese Asian regions. The median duration of edoxaban use was 24.0 months, with a median of 19.2 months in Japan, 24.0 months in Europe, and 23.3 months in non‐Japanese Asian regions.

### 3.3 Baseline Demographics and Clinical Characteristics

The baseline demographics and clinical characteristics are shown in Table 1. Overall, patients had a mean ± SD age of 73.6 ± 9.7 years, with patients in Japan (74.2 ± 10.0 years) being older than those in Europe (73.6 ± 9.5 years) or other non‐Japanese Asian regions (71.7 ± 9.7 years). Across all regions, most patients were male (58.1%). Compared with patients from Europe, patients from Japan and non‐Japanese Asian regions weighed less (mean ± SD body weight; Europe, 80.9 ± 17.2 kg; Japan, 60.1 ± 12.8 kg; non‐Japanese Asian regions, 65.9 ± 12.5 kg). At baseline, the median (IQR) CHA_2_DS_2_‐VASc score overall and in each region was 3.0 (2.0–4.0). The median (IQR) for modified HAS‐BLED score overall and in each region was 3.0 (2.0–4.0).

**Table 1:** Baseline demographics and clinical characteristics of patients with 2‐year follow‐up.

	Global *N* = 26 805	Europe *n* = 13 164^a^	Japan *n* = 10 342	Non‐Japanese Asian regions *n* = 3299^a^	*p* value^b^
Age, years	73.6 ± 9.7	73.6 ± 9.5	74.2 ± 10.0	71.7 ± 9.7	< 0.0001
Median (IQR)	75.0 (68.0–80.0)	75.0 (68.0–80.0)	75.0 (68.0–81.0)	72.0 (66.0–79.0)	
Sex, male, *n* (%)	15 573 (58.1)	7461 (56.7)	6168 (59.6)	1944 (58.9)	< 0.0001
Weight, kg	72.2 ± 18.1	80.9 ± 17.2	60.1 ± 12.8	65.9 ± 12.5	< 0.0001
BMI, kg/m^2^	26.4 ± 5.0	28.1 ± 5.1	23.6 ± 3.8	25.1 ± 3.8	< 0.0001
CrCl CG, mL/min	69.2 ± 28.6	74.3 ± 30.3	63.8 ± 25.7	62.3 ± 23.9	< 0.0001
Serum creatinine, mg/dL	0.97 ± 0.44	1.02 ± 0.32	0.88 ± 0.26	1.04 ± 0.94	< 0.0001
Comorbidities, *n* (%)					
COPD	1510 (5.6)	1206 (9.2)	147 (1.4)	157 (4.8)	< 0.0001
Diabetes mellitus	6299 (23.5)	2881 (21.9)	2431 (23.5)	987 (29.9)	< 0.0001
Hypertension	20 023 (74.7)	10 155 (77.1)	7477 (72.3)	2391 (72.5)	< 0.0001
Ischemic stroke	2918 (10.9)	697 (5.3)	1778 (17.2)	443 (13.4)	< 0.0001
TIA	832 (3.1)	449 (3.4)	309 (3.0)	74 (2.2)	0.002
Heart failure^c^	5275 (19.7)	2042 (15.5)	2795 (27.0)	438 (13.3)	< 0.0001
Myocardial infarction	1010 (3.8)	567 (4.3)	382 (3.7)	61 (1.8)	< 0.0001
Peripheral artery disease	626 (2.3)	432 (3.3)	170 (1.6)	24 (0.7)	< 0.0001
Type of AF, *n* (%)					
Paroxysmal	13 341 (49.8)	7083 (53.9)	4769 (46.1)	1489 (45.2)	
Persistent	5859 (21.9)	3175 (24.2)	1916 (18.5)	768 (23.3)	< 0.0001
Persistent and permanent	7575 (28.3)	2881 (21.9)	3656 (35.4)	1038 (31.5)	
History of major bleeding, *n* (%)	445 (1.7)	118 (0.9)	252 (2.4)	75 (2.3)	< 0.0001
Intracranial, *n* (%)	334 (1.2)	62 (0.5)	230 (2.2)	42 (1.3)	< 0.0001
Major GI, *n* (%)	75 (0.3)	35 (0.3)	16 (0.2)	24 (0.7)	< 0.0001
CHA_2_DS_2_‐VASc					
Mean ± SD	3.3 ± 1.5	3.2 ± 1.4	3.4 ± 1.6	3.2 ± 1.5	< 0.0001
Median (IQR)	3.0 (2.0–4.0)	3.0 (2.0–4.0)	3.0 (2.0–4.0)	3.0 (2.0–4.0)	
HAS‐BLED					
Mean ± SD	2.7 ± 1.1	2.5 ± 1.0	3.0 ± 1.1	2.6 ± 1.0	< 0.0001
Median (IQR)	3.0 (2.0–3.0)	3.0 (2.0–3.0)	3.0 (2.0–4.0)	3.0 (2.0–3.0)	
AF‐related treatment before baseline					
Previous VKA, *n* (%)	NA	2457 (18.7)	1325 (12.8)	615 (18.6)	NA
Previous DOAC, *n* (%)	NA	1175 (8.9)	1139 (11.0)	847 (25.7)	NA
Concomitant antiplatelet, *n* (%)	NA	2913 (22.1)	1595 (15.4)	868 (26.3)	NA
Edoxaban 60 mg, *n* (%)^d^	13 918 (54.5)	9617 (76.0)	2693 (27.9)	1608 (49.6)	< 0.0001
Dosing according to label, *n* (%)	17 449 (68.3)	9116 (72.0)	6193 (64.1)	2140 (65.9)	< 0.0001
Duration of edoxaban use from baseline to 2 years, months	19.3 ± 7.2	21.0 ± 6.5	16.9 ± 7.5	19.7 ± 7.1	< 0.0001
Median (IQR)	24.0 (14.0–24.0)	24.0 (23.7–24.0)	19.2 (11.7–24.0)	23.3 (19.4–24.0)	
Permanent edoxaban discontinuation from baseline to 2 years, *n* (%)	3530 (13.2)	2119 (16.1)	830 (8.0)	581 (17.6)	< 0.0001

*Note:* Data shown as mean ± SD unless otherwise indicated.

Abbreviations: AF, atrial fibrillation; BMI, body mass index; CG, Cockcroft–Gault; CHA_2_DS_2_‐VASc, Congestive heart failure, Hypertension, Age ≥ 75 years (doubled), Diabetes mellitus, Stroke (doubled), Vascular disease, Age 65–74 years, Sex category (female); COPD, chronic obstructive pulmonary disease; CrCl, creatinine clearance; DOAC, direct oral anticoagulant; GI, gastrointestinal; HAS‐BLED, Hypertension, Abnormal renal/liver function, Stroke, Bleeding history or predisposition, Labile international normalized ratio, Elderly, Drugs/alcohol concomitantly; IQR, interquartile range; MI, myocardial infarction; NA, not applicable; SD, standard deviation; TIA, transient ischemic attack; VKA, vitamin K antagonist.

^a^ Patient numbers for Europe and non‐Japanese Asian regions are higher than in the 1‐year follow‐up because some patients did not attend the 1‐year follow‐up but were seen during the second year or because an event was reported.

^b^ *p* values are based on a *χ* ^2^ test for categorical data and on the Kruskal–Wallis test for continuous variables to determine the effect of region.

^c^ A medical history of heart failure required the fulfillment of one of the following criteria: Documented congestive heart failure or, if congestive heart failure was not documented, then documentation of ischemic cardiomyopathy; ejection fraction < 40%; frequent dyspnea (≥ 1/day) without COPD and with documented severe valvular heart disease, coronary heart disease post‐MI, valve replacement, or hypertension treated with ≥ 3 drugs.

^d^ For edoxaban dosage category and for treatment according to label, percentages are based on the number of patients with an edoxaban dose of either 30 or 60 mg. For everything else, percentages are based on the *N* in the column headers, that is, missing and unknown patients are not excluded from the denominator when calculating the percentages.

The percentage of patients with a history of ischemic stroke (17.2%) or heart failure (HF; 27.0%) was highest in Japan. History of ischemic stroke was lowest in Europe (5.3%), and history of HF was lowest in non‐Japanese Asian regions (13.3%). Patients from Europe had the highest burden of comorbidities, including hypertension (77.1%), chronic obstructive pulmonary disease (COPD; 9.2%), MI (4.3%), and peripheral artery disease (3.3%), compared with patients in Japan and non‐Japanese Asian regions. Diabetes mellitus was highest in patients from non‐Japanese Asian regions (29.9%) versus patients in Japan (23.5%) or Europe (21.9%). Patients from Japan were more likely to have had a history of major bleeding events (2.4%) compared with patients from Europe and non‐Japanese Asian regions. This higher proportion was mainly due to a higher proportion of patients in Japan having a history of intracranial hemorrhage (2.2%; Table 1).

### 3.4 Annualized Clinical Events Over the 2‐Year Follow‐Up (Months 1–24)

**Figure 1:**
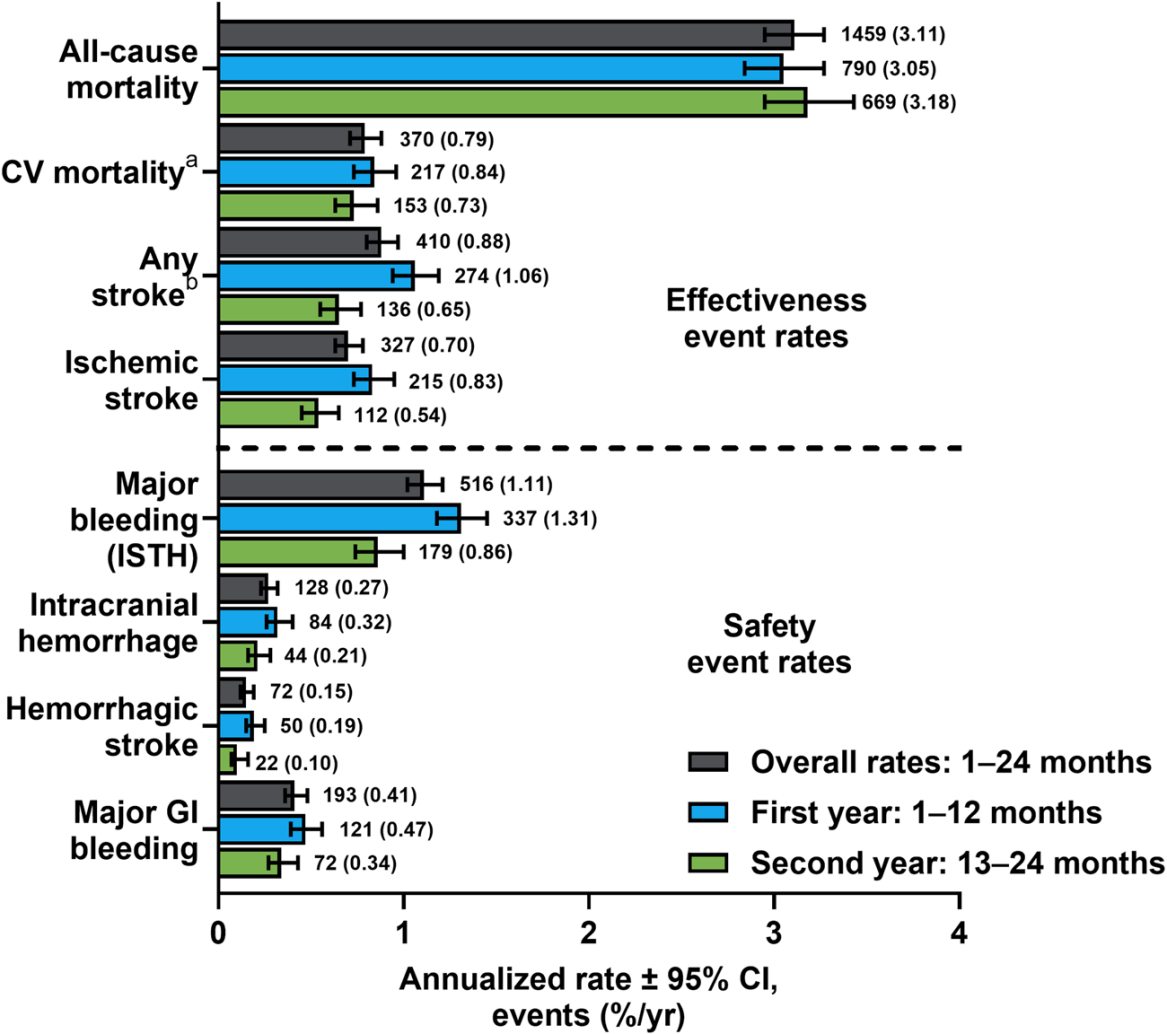
Global effectiveness and safety annualized clinical event rates overall as well as in the first year (months 1–12) and second year (months 13–24). ^a^CV mortality is defined as deaths due to CV‐related reasons plus deaths where there was a bleeding event with fatal outcome or where any stroke, TIA, SEE, PE, MI, VTE, or major bleeding occurred within 30 days before death and the death reason was missing or unknown. For all regions, it is censored by 730 days, study discontinuation, or last follow‐up, whichever comes first. ^b^Includes patients (*n* = 15) with unknown stroke type. CI, confidence interval; CV, cardiovascular; GI, gastrointestinal; ISTH, International Society on Thrombosis and Haemostasis; MI, myocardial infarction; PE, pulmonary embolism; SEE, systemic embolic event; TIA, transient ischemic attack; VTE, venous thromboembolism.

In the Global ETNA‐AF population, the annualized rates of any stroke and other thromboembolic events were low over the 2‐year follow‐up (months 1–24; Figure 1). The annualized event rate (95% CI) from months 1 to 24 for any stroke was 0.88%/year (0.80%–0.97%/year) including 0.70%/year (0.63%–0.78%/year) for ischemic and 0.15%/year (0.12%–0.19%/year) for hemorrhagic stroke. Over the 2‐year follow‐up, the annualized event rate (95% CI) of all‐cause mortality was 3.11%/year (2.95%–3.27%/year) and 0.79%/year (0.71%–0.87%/year) for CV mortality. CV deaths of patients from Japan occurring after study discontinuation or last follow‐up were not part of the primary analysis. Following a conservative approach and including these events raises the global annual CV mortality from 0.79%/year to 0.91%/year. Bleeding annualized event rates were also low at the 2‐year follow‐up; the annualized event rate (95% CI) for major bleeding was 1.11%/year (1.02%–1.21%/year) with annualized event rates (95% CI) of 0.27%/year (0.23%–0.32%/year) for intracranial hemorrhage and 0.41%/year (0.36%–0.48%/year) for major GI bleeding.

**Figure 2:**
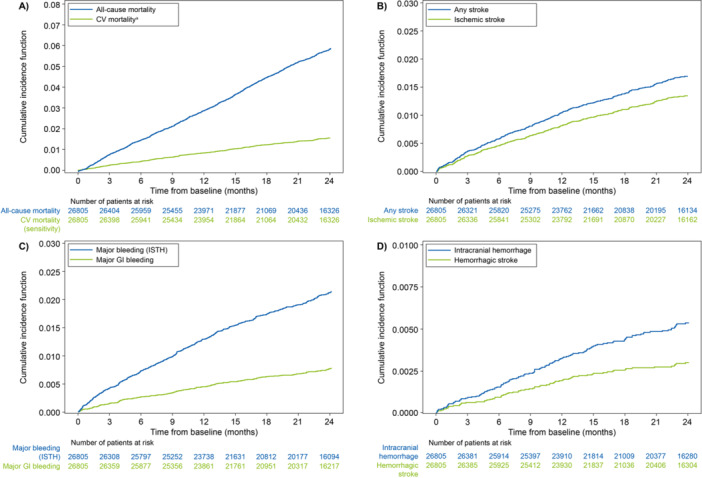
Cumulative incidence function curves from 0 to 24 months for (A) all‐cause mortality and CV mortality, (B) any stroke and ischemic stroke, (C) major bleeding and major GI bleeding, and (D) intracranial hemorrhage and hemorrhagic stroke. ^a^CV mortality is defined as deaths due to CV‐related reasons plus deaths where there was a bleeding event with fatal outcome or where any stroke, TIA, SEE, PE, MI, VTE, or major bleeding occurred within 30 days before death and the death reason was missing or unknown. For all regions, it is censored by 730 days, study discontinuation, or last follow‐up, whichever comes first. CV, cardiovascular; GI, gastrointestinal; ISTH, International Society on Thrombosis and Haemostasis; MI, myocardial infarction; PE, pulmonary embolism; SEE, systemic embolic event; TIA, transient ischemic attack; VTE, venous thromboembolism.

Kaplan–Meier CIF curves for the Global ETNA‐AF population are presented in Figure 2A–D. CIF curves of all‐cause mortality and CV mortality showed a stable accrual of events from 0 months to 24 months (Figure 2A). CIF curves for any stroke and ischemic stroke (Figure 2B), major bleeding and major GI bleeding (Figure 2C), and ICH and hemorrhagic stroke (Figure 2D) showed that the accrual rate slowed between 0 and 24 months.

### 3.5 Regional Annualized Clinical Events Over the 2‐Year Follow‐Up (Months 1–24)

**Figure 3:**
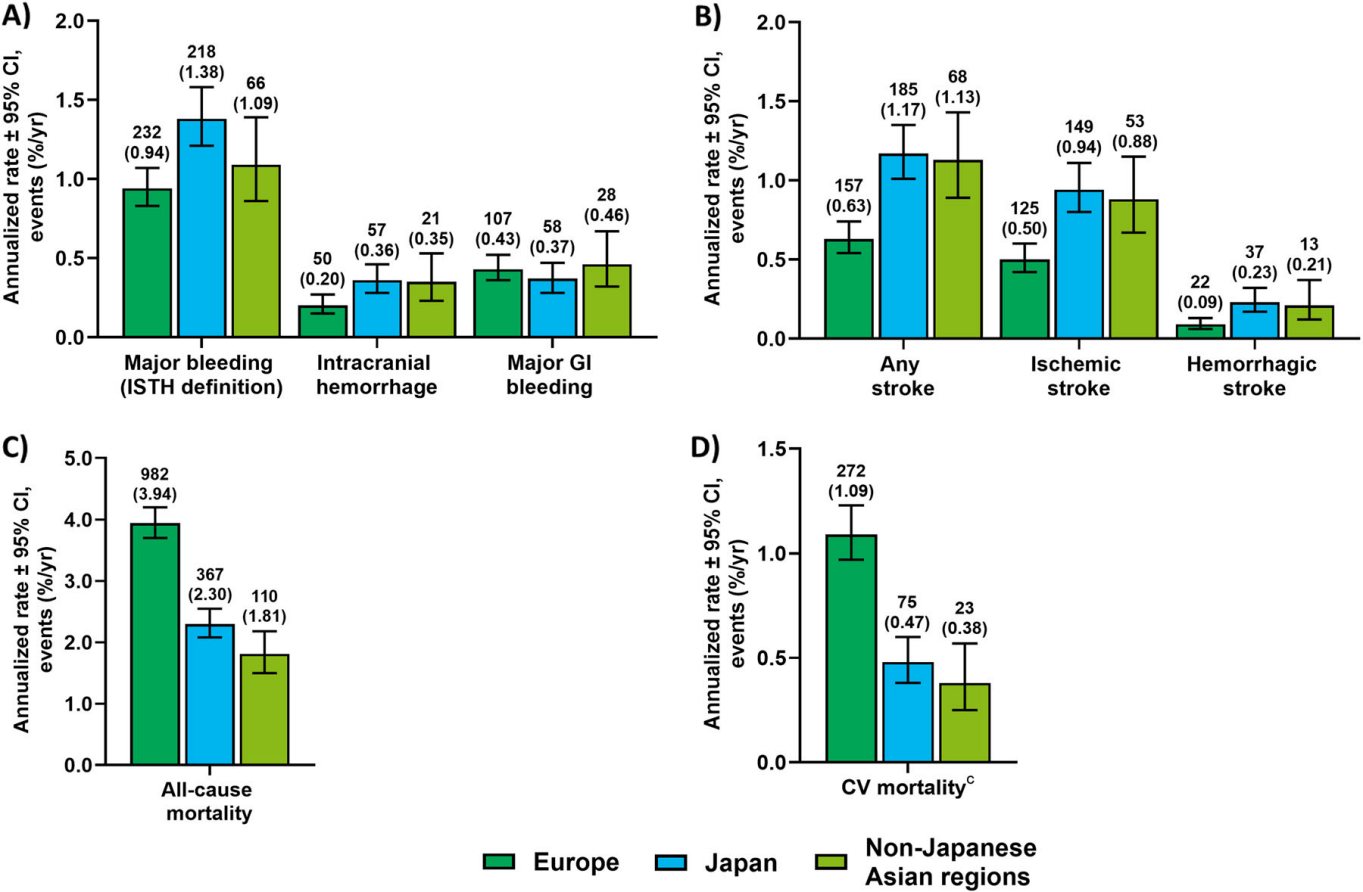
Annualized clinical event rates^a^ for (A) major bleeding, (B) any stroke, (C) all‐cause mortality, and (D) CV mortality in Europe, Japan, and non‐Japanese Asian^b^ regions over the 2‐year follow‐up (months 1–24). ^a^Bar graphs show the annualized rate ± 95% CI. Clinical event counts (with the annualized rate shown in parentheses below them) are shown above each bar for each region. ^b^Non‐Japanese Asian regions include Hong Kong, South Korea, Taiwan, and Thailand. ^c^CV mortality is defined as deaths due to CV‐related reasons plus deaths where there was a bleeding event with fatal outcome or where a stroke, TIA, SEE, PE, MI, VTE, or major bleeding occurred within 30 days before death and the death reason was missing or unknown. For all regions, it is censored by 730 days, study discontinuation, or last follow‐up, whichever comes first. CI, confidence interval; CV, cardiovascular; GI, gastrointestinal; ISTH, International Society on Thrombosis and Haemostasis; MI, myocardial infarction; PE, pulmonary embolism; SEE, systemic embolic event; TIA, transient ischemic attack; VTE, venous thromboembolism.

There were regional differences for both effectiveness (any stroke and all‐cause mortality) and safety (major bleeding) annualized event rates over the 2‐year follow‐up (months 1–24). Annualized event rates for any stroke from months 1 to 24 were numerically highest in Japan (1.17%/year; 95% CI, 1.01%–1.35%/year) and lowest in Europe (0.63%/year; 95% CI, 0.54%–0.74%/year). Over the 2‐year follow‐up, all‐cause mortality was numerically highest in Europe (3.94%/year; 95% CI, 3.70%–4.20%/year) and lowest in non‐Japanese Asian regions (1.81%/year; 95% CI, 1.50%–2.18%/year). Adding deaths after study discontinuation or last follow‐up of Japanese patients raises the annual CV mortality in Japan from 0.47%/year to 0.82%/year, i.e., between those for non‐Japanese Asian countries (0.38%/year) and Europe (1.09%/year). Annualized event rates for major bleeding from months 1 to 24 were numerically highest in Japan (1.38%/year; 95% CI, 1.21%–1.58%/year) and lowest in Europe (0.94%/year; 95% CI, 0.83%–1.07%/year; Figure 3).

**Figure 4:**
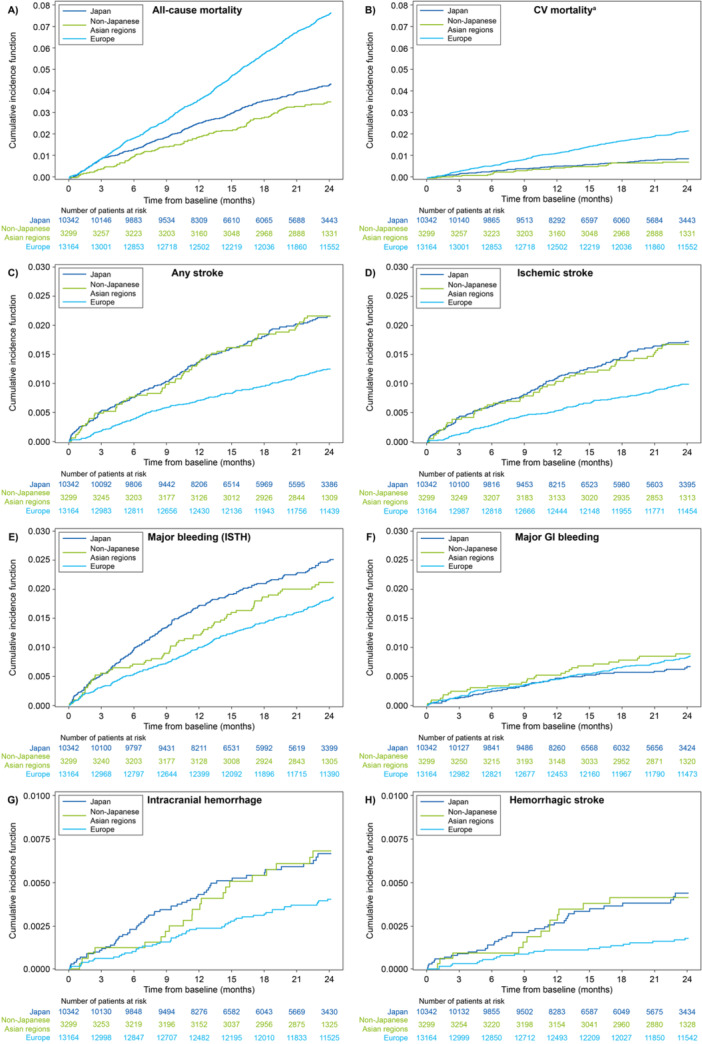
Cumulative incidence function curves from months 0 to 24 by region for (A) all‐cause mortality, (B) CV mortality, (C) any stroke, (D) ischemic stroke, (E) major bleeding, (F) major GI bleeding, (G) intracranial hemorrhage, and (H) hemorrhagic stroke. ^a^CV mortality is defined as death due to CV‐related reasons plus deaths where there was a bleeding event with fatal outcome or where any stroke, TIA, SEE, PE, MI, VTE, or major bleeding occurred within 30 days before death and the death reason was missing or unknown. For all regions, it is censored by 730 days, study discontinuation, or last follow‐up, whichever comes first. CV, cardiovascular; GI, gastrointestinal; ISTH, International Society on Thrombosis and Haemostasis; MI, myocardial infarction; PE, pulmonary embolism; SEE, systemic embolic event; TIA, transient ischemic attack; VTE, venous thromboembolism.

CIF curves by region are shown in Figure 4A–H. The accrual for all‐cause mortality events (Figure 4A) was similar between Europe and Japan over the first 3 months. After the first 3 months, there was a stable accrual of all‐cause mortality events in Europe and Japan, though the rate of accrual was slower in Japan. In non‐Japanese Asian regions, the accrual rate of all‐cause death was slower in the first 3 months relative to Japan and Europe. However, from 3 to 24 months this accrual rate was similar to that in Japan and remained slower than that in Europe. There were stable accrual rates for CV death in all three regions with the steepest rate in Europe, followed by Japan and non‐Japanese Asian regions (Figure 4B). CIF curves for Japan and non‐Japanese Asian regions compared with Europe had steeper slopes for any stroke (Figure 4C) and ischemic stroke (Figure 4D) with the accrual rate slowing between 0 and 24 months. CIF curves for major bleeding had the steepest slope in Japan compared with Europe or non‐Japanese Asian regions (Figure 4E). The highest incidence of major bleeding was observed in Japan, though the accrual rate incrementally slowed up to 24 months, whereas in non‐Japanese Asian regions the accrual rate varied over time. The lowest incidence of major bleeding was observed in Europe, and there was a stable accrual of major bleeding events over 24 months. For major GI bleeding (Figure 4F), the CIFs were similar between regions. For ICH (Figure 4G) and hemorrhagic stroke (Figure 4H), the accrual rates were similar between Japan and non‐Japanese Asian regions for the first 3 months. At 24 months, the CIF was highest in Japan and non‐Japanese Asian regions, but in Japan the accrual rate incrementally slowed down between 0 and 24 months, whereas in non‐Japanese Asian regions the accrual rate varied over time. The rates of accrual were lowest in Europe, which had a stable accrual rate between 0 and 24 months. Among patients who were alive and event‐free at 1 year, CIFs for all‐cause mortality (Figure S1A) and CV mortality (Figure S1B) were similar from 13 to 24 months between Japan and non‐Japanese Asian regions, whereas the CIF for Europe remained higher than that for either Japan or non‐Japanese Asian regions. For all other endpoints, the three regions had similar cumulative incidence rates between 13 and 24 months (Figure S1C–H).

### 3.6 Global Annualized Clinical Event Rates in the First (Months 1–12) and Second Year (Months 13–24)

In this conditional survival analysis, there were numerical differences in the annualized event rates for effectiveness and safety observed in the first year (months 1–12) and the second year (months 13–24). Annualized event rates (95% CI) in the first year and second year were 1.06%/year (95% CI, 0.94%–1.19%/year) and 0.65%/year (95% CI, 0.55%–0.77%/year) for any stroke as well as 0.83%/year (0.73%–0.95%/year) and 0.54%/year (0.45%–0.65%/year) for ischemic stroke (Figure 1). The annualized event rates (95% CI) in the first and second year were 3.05%/year (2.84%–3.27%/year) and 3.18%/year (2.95%–3.43%/year) for all‐cause death and 0.84%/year (0.73%–0.96%/year) and 0.73%/year (0.63%–0.86%/year) for CV death. The annualized event rates (95% CI) in the first and second year were 1.31%/year (1.18%–1.45%/year) and 0.86%/year (0.74%–1.00%/year) for major bleeding (Figure 1). Additionally, the annualized event rates (95% CI) in the first and second year were 0.32%/year (0.26%–0.40%/year) and 0.21%/year (0.16%–0.28%/year) for intracranial hemorrhage as well as 0.47%/year (0.39%–0.56%/year) and 0.34%/year (0.27%–0.43%/year) for major GI bleeding. For the sensitivity analysis, if repeated events were taken into consideration, annualized event rates remained consistent in the first and second year (Figure S2).

## 4 Discussion

This analysis presents the 2‐year follow‐up from the Global ETNA‐AF program, the largest prospective, noninterventional program evaluating the effectiveness and safety of a single DOAC in 26 805 patients with AF across Europe (49.1%), Japan (38.6%), and non‐Japanese Asian regions (12.3%), including Hong Kong, South Korea, Taiwan, and Thailand. Notably, 50.9% of included patients were from Asian regions. Clinical annualized event rates over the observational period of 2 years for any stroke and major bleeding were low among patients receiving edoxaban at baseline for the prevention of stroke in routine clinical practice, consistent with findings reported at the 1‐year follow‐up [8]. This 2‐year analysis expands on the previously reported 1‐year results that included slightly fewer patients in the observed countries and no patients from Hong Kong or Thailand [8].

Over 2 years, all‐cause mortality was numerically higher in Europe compared with Japan and non‐Japanese Asian regions (Europe, 3.94%/year; Japan, 2.30%/year; non‐Japanese Asian regions, 1.81%/year). Similarly, CV mortality from months 1 to 24 was also numerically higher in Europe compared with Japan and non‐Japanese Asian regions (Europe, 1.09%/year; Japan, 0.47%/year; non‐Japanese Asian regions, 0.38%/year). The highest burden of CV comorbidities at baseline, including hypertension, MI, peripheral artery disease, and COPD, was observed in the European population, whereas the proportion of patients from Japan and non‐Japanese Asian regions with these comorbidities was numerically lower. This burden of comorbidities may, in part, be explained by the higher body mass index (BMI) in European patients versus patients in Japan and non‐Japanese Asian regions. In general, patients from Asian countries, particularly Japan, have a lower body weight than their Western counterparts [17]. A higher BMI is associated with an increased risk of developing CV comorbidities, which may contribute to the differences in all‐cause and CV mortality between regions in this analysis [18, 19, 20, 21].

Differences in other baseline patient characteristics across regions may also help to explain the higher annualized event rates of any stroke and major bleeding over the 2‐year follow‐up observed in Japan compared with Europe and non‐Japanese Asian regions. Patients from Japan and non‐Japanese Asian regions versus Europe were older and more likely to have long‐standing persistent and permanent AF, as well as histories of ischemic stroke and major bleeding, including intracranial hemorrhage. History of major bleeding, advanced age, or lower body weight increase the risk of future thromboembolic and bleeding events and are associated with adverse clinical outcomes in patients with AF [22, 23, 24]. Such trends may contribute to the higher annualized rates of any stroke, including ischemic stroke, and major bleeding events observed in this study [25].

Although it is difficult to compare real‐world studies with randomized controlled trials directly, there are some differences in annualized clinical event rates observed in this large, prospective, noninterventional study compared with the randomized controlled ENGAGE AF‐TIMI 48 trial. Whereas the Global ETNA‐AF program includes patients from Europe, Japan, and other non‐Japanese Asian regions, the ENGAGE AF‐TIMI 48 trial included patients from North America, Latin America, Western Europe, Eastern Europe, the Asia‐Pacific region, and South Africa. The present cohort reported lower annualized event rates of ischemic stroke than ENGAGE AF‐TIMI 48 (ETNA‐AF, 0.7%/year vs. ENGAGE AF‐TIMI 48, 1.25%/year), major bleeding (ETNA‐AF, 1.1%/year vs. ENGAGE AF‐TIMI 48, 2.75%/year), and major GI bleeding (ETNA‐AF, 0.4%/year vs. ENGAGE AF‐TIMI 48, 1.51%/year) [2]. The annualized event rates of intracranial hemorrhage and hemorrhagic stroke were similar [2]. This may be due to the very systematic and granular collection of outcome events in a phase 3 trial. In addition, the Global ETNA‐AF program enrolled a broader range of patients with AF who were healthier and who had a lower overall risk of stroke than those in the ENGAGE AF‐TIMI 48 trial, including a less frequent history of HF (27.4% vs. 58.2%), hypertension (74.7% vs. 93.7%), and diabetes (23.5% vs. 36.4%) [2]. Regional differences in the rates of stroke/SEEs and major bleeding were also noted in the ENGAGE AF‐TIMI 48 trial, with patients on edoxaban in East Asia versus Japan having lower rates of stroke/SEEs (East Asia, 1.17%/year; Japan, 1.47%/year) and major bleeding (East Asia, 2.23%/year; Japan, 3.38%/year) [26]. In this Global ETNA‐AF program, a numerically lower annualized event rate over months 1 to 24 of any stroke and major bleeding were reported in patients in non‐Japanese Asian regions versus Japan, suggesting regional differences may impact stroke and major bleeding event rates.

The low annualized event rates over months 1 to 24 of any stroke and major bleeding documented in the Global ETNA‐AF population are consistent with those reported in other real‐world population‐based studies [12, 27]. In comparing results from the GARFIELD‐AF registry 2‐year follow‐up and GLORIA‐AF registry 2‐year follow‐up, which evaluated risk of any stroke in patients with newly diagnosed AF, this ETNA‐AF 2‐year follow‐up reported similarly low annualized rates of any stroke (GARFIELD‐AF, 1.3%/year; GLORIA‐AF, 0.6%/year; ETNA‐AF, 0.9%/year) and major bleeding (GARFIELD‐AF, 0.7%/year; GLORIA‐AF, 1.1%/year; ETNA‐AF, 1.1%/year) [12, 27]. Patients in the Global ETNA‐AF program were generally older and from a more diverse population than those in GLORIA‐AF or GARFIELD‐AF, but other key baseline patient characteristics were similar across studies [12, 27]. Taken together, these findings reaffirm the effectiveness and safety of edoxaban in patients with AF in routine clinical practice.

Overall, the annualized rates for ischemic stroke and major bleeding in this real‐world population over the 2‐year (1–24 months) follow‐up were low (< 2%), supporting the results from the 1‐year follow‐up. The annualized event rates of any stroke and major bleeding in the second year (months 13–24) were slightly lower compared with the first year (months 1–12) [8]. Of note, the first 3 to 6 months of oral anticoagulant treatment constitute a high‐risk period for thromboembolic and bleeding complications [27], which possibly impacted the annualized event rates reported in the first year. A slight increase in the annualized event rate of global all‐cause mortality was observed from the first year (3.05%/year) to the second year (3.18%/year). However, all‐cause mortality decreased from the first year to the second year in Japan (annualized event rate in the first year vs. second year: 2.57%/year vs. 1.87%/year) and in the non‐Japanese Asian regions (annualized event rate in the first year vs. second year: 1.92%/year vs. 1.68%/year), and increased in Europe (annualized event rate in the first year vs. second year: 3.70%/year vs. 4.20%/year). These differences in temporal trends across regions may be due to the European cohort having a higher burden of comorbidities compared with Japan and non‐Japanese Asian regions.

The current study has limitations. In this conditional survival analysis, the annualized event rates in the second year (months 13–24) may be underestimated given that patients were conditionally included based on being event‐free during the first year (1–12 months). To help account for this potential underestimation, repeated events were taken into consideration in the sensitivity analysis and the decline in annualized event rates from the first to second year remained consistent. Additionally, it should be noted that the patient cohort during the second year is slightly different than the cohort during the first year (e.g., premature termination of patients included in the first‐year analysis and treatment discontinuation); however, without baseline characteristics at the start of the second year, this difference in patient cohorts could not be quantified. Furthermore, no comparator arm was included in this noninterventional, observational study, so no comparisons can be drawn regarding the effectiveness and safety of edoxaban relative to other oral anticoagulants.

## 5 Conclusion

Annualized event rates over the 2‐year follow‐up for any stroke and major bleeding were low in patients with AF receiving edoxaban at baseline. Regional differences in baseline patient disease history and comorbidities may partially explain differences in the incidence of major bleeding, any stroke, and all‐cause death annualized event rates.

## Author Contributions

**Raffaele De Caterina:** conceptualization, methodology, validation, investigation, writing – original draft preparation, writing – review and editing, visualization, supervision, project administration. **Paulus Kirchhof:** conceptualization, methodology, validation, investigation, writing – original draft preparation, writing – review and editing, visualization, supervision, project administration. **Martin Unverdorben:** methodology, validation, writing – original draft preparation, writing – review and editing, visualization, supervision, project administration. **Cathy Chen:** methodology, validation, writing – original draft preparation, writing – review and editing, visualization, supervision, project administration. **Eue‐Keun Choi:** methodology, investigation, writing – original draft preparation, writing – review and editing, visualization, project administration. **Yukihiro Koretsune:** methodology, investigation, writing – original draft preparation, writing – review and editing, visualization, project administration. **Chun‐Chieh Wang:** methodology, investigation, writing – original draft preparation, writing – review and editing, visualization, project administration. **Takeshi Yamashita:** methodology, investigation, writing – original draft preparation, writing – review and editing, visualization, project administration. **Ladislav Pecen:** methodology, validation, formal analysis, data curation, writing – original draft preparation, writing – review and editing, visualization. **Peter Bramlage:** writing – original draft preparation, writing – review and editing, visualization. **Doralisa Morrone:** writing – review and editing, visualization. All authors have read and agreed to the published version of the manuscript.

## Consent

All ETNA‐AF participants provided written consent before enrollment; patients who did not or were part of a simultaneous interventional study were excluded from participation.

## Ethics Statement

The responsible ethics committees and institutional review boards approved the study protocols, except in Japan, where such approval is not required for this study type. The program complied with the Declaration of Helsinki and the International Conference on Harmonisation of Good Clinical Practice standards.

## Conflicts of Interest

R.D.C. declares fees, honoraria, and research funding from Amgen, Bayer, Boehringer Ingelheim, Bristol Myers Squibb/Pfizer, Daiichi Sankyo, Menarini, Merck, Novartis, Portola, Roche, and Sanofi‐Aventis. M.U. and C.C. are employees of Daiichi Sankyo. E.‐K.C. declares research grants or speaking fees from Abbott, Bayer, Biosense Webster, BMS/Pfizer, Chong Kun Dang, Daewoong Pharmaceutical, Daiichi Sankyo, DeepQure, Dreamtech, Jeil Pharmaceutical, Medtronic, Samjinpharm, Samsung Electronics, Seers Technology, and Skylabs. Y.K. declares honoraria from Boehringer Ingelheim, Bristol Myers Squibb/Pfizer, and Daiichi Sankyo. D.M. declares fees, honoraria, and research funding from Daiichi Sankyo. L.P. declares to have received funding for the statistics of this report from Daiichi Sankyo. P.B. declares to have received funding for the writing of this report from Daiichi Sankyo. C.C.W. declares honoraria from AstraZeneca, Bayer, Boehringer Ingelheim, Daiichi Sankyo, Novartis, and Pfizer. T.Y. declares fees, honoraria, and research funding from Bayer, Bristol Myers Squibb, Daiichi Sankyo, Ono Pharmaceutical, and Toa Eiyo. P.K. declares research support for basic, translational, and clinical research projects from the British Heart Foundation, European Union, German Centre for Cardiovascular Research, and Medical Research Council (UK); and funding and honoraria from several drug and device companies active in atrial fibrillation in the past, but not in the last 3 years.

## Supporting information

Supporting information.

## Data Availability

Data will not be made available from this analysis because the study is still ongoing.
