# Supplementary material for: Two‐Year Follow‐Up of Patients With Atrial Fibrillation Receiving Edoxaban in Routine Clinical Practice: Results From the Global ETNA‐AF Program
Source: Clin Cardiol. 2025 Feb 27;48(3):e70091. doi: 10.1002/clc.70091 (PMC11885795; doi:10.1002/clc.70091)
Supplement: Supplementary file 1 — Supporting information. [file CLC-48-e70091-s001.docx]

**Supplemental Material**

**Two-year follow-up of patients with atrial fibrillation receiving edoxaban in routine clinical practice: Results from the Global ETNA-AF program**

**Short title:** ETNA-AF 2-year follow-up

Raffaele De Caterina, MD, PhD^1, 2,15^; Martin Unverdorben, MD, PhD^3,15^; Cathy Chen, MD^3,15^; Eue-Keun Choi, MD, PhD^4,15^; Yukihiro Koretsune, MD, PhD^5,15^; Doralisa Morrone, MD, PhD^6,15^; Ladislav Pecen, PhD^7, 8,15^; Peter Bramlage, MD, PhD^9,15^; Chun-Chieh Wang, MD^10,15^; Takeshi Yamashita, MD, PhD^11,15^; Paulus Kirchhof, MD, PhD^12–15^

^1^Chair of Cardiology, University of Pisa and Cardiology Division, University of Pisa, Pisa, Italy

^2^Fondazione Villa Serena per la Ricerca, Città Sant'Angelo, Italy

^3^Daiichi Sankyo, Inc., Basking Ridge, USA

^4^Department of Internal Medicine, Seoul National University College of Medicine and Seoul National University Hospital, Seoul, Republic of Korea

^5^National Hospital Organization Osaka National Hospital, Osaka, Japan

^6^Division of Cardiology, Department of Surgical, Medical and Molecular Pathology and Critical Care Medicine, University of Pisa, Pisa, Italy

^7^Institute of Computer Science of the Czech Academy of Sciences, Prague, Czech Republic

^8^Medical Faculty, Charles University, Pilsen, Czech Republic

^9^Institute for Pharmacology and Preventive Medicine, Cloppenburg, Germany

^10^Department of Cardiology, Chang Gung Memorial Hospital, Chang Gung University, Taoyuan, Taiwan

^11^Department of Cardiovascular Medicine, Cardiovascular Institute, Tokyo, Japan

^12^Center for Cardiovascular Research, University of Birmingham and SWBH and UHB NHS Trusts, Birmingham, UK

^13^Department of Cardiology, University Heart and Vascular Center Hamburg, Hamburg, Germany

^14^German Center for Cardiovascular Research (DZHK) Partner Site Hamburg/Kiel/Lübeck, Lübeck, Germany

^15^This author takes responsibility for all aspects of the reliability and freedom from bias of the data presented and their discussed interpretation

**Correspondence**

Professor Raffaele De Caterina

Department of Cardiology, University of Pisa, Pisa University Hospital

Via Paradisa 2, 56125 Pisa, Italy

Email: [raffaele.decaterina@unipi.it](mailto:raffaele.decaterina@unipi.it)

**
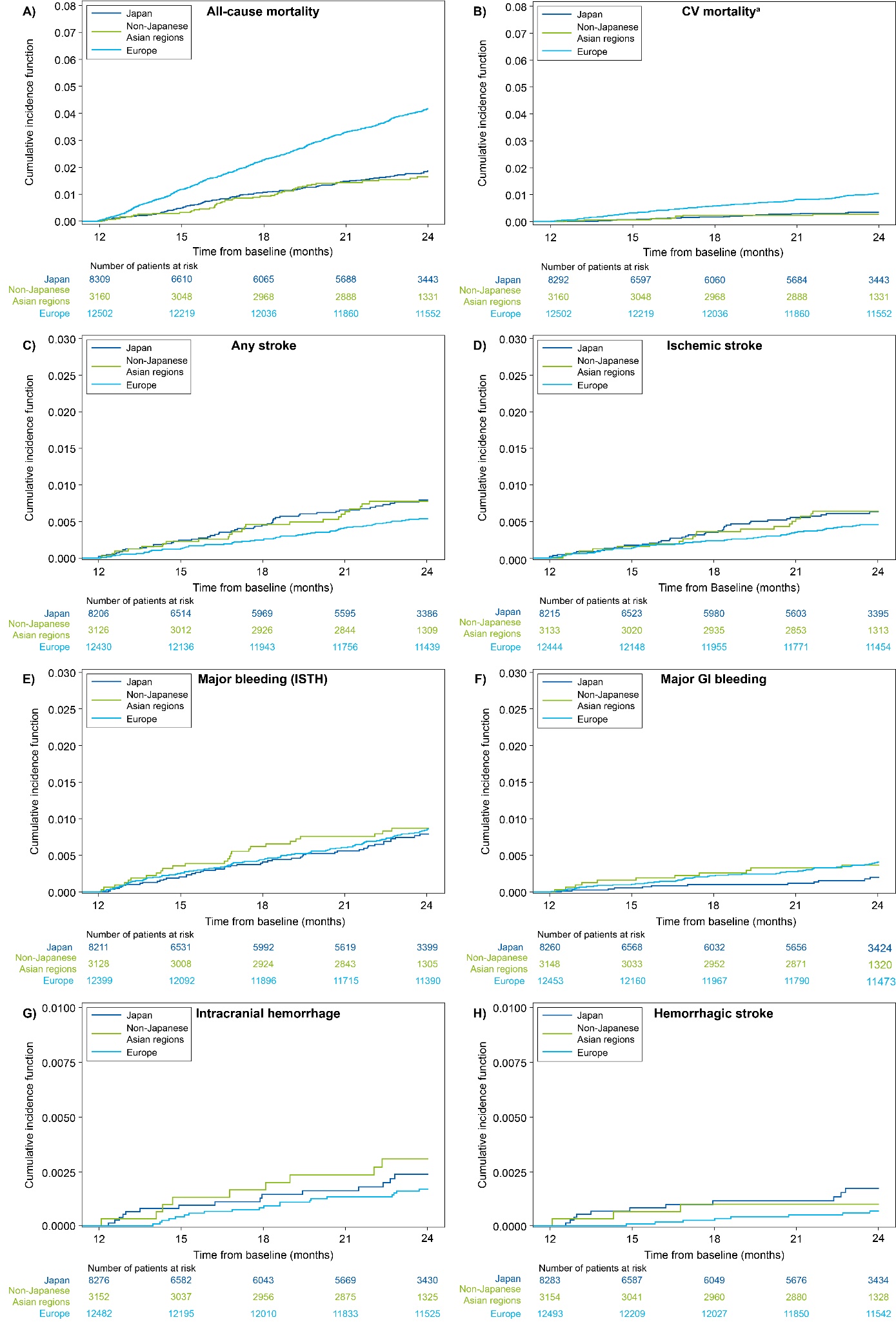
**

**SUPPLEMENTAL FIGURE 1** Cumulative incidence function curves for months 12 to 24 by region of A) all-cause mortality, B) CV mortality, C) any stroke, D) ischemic stroke, E) major bleeding, F) major GI bleeding, G) intracranial hemorrhage, and H) hemorrhagic stroke.

^a^CV mortality is defined as deaths due to CV-related reasons plus deaths where there was a bleeding event with fatal outcome or where any stroke, TIA, SEE, PE, MI, VTE, or major bleeding occurred within 30 days before death and the death reason was missing or unknown. For all regions, it is censored by 730 days, study discontinuation, or last follow-up, whichever comes first. CV, cardiovascular; GI, gastrointestinal; ISTH, International Society on Thrombosis and Haemostasis; MI, myocardial infarction; PE, pulmonary embolism; SEE, systemic embolic event; TIA, transient ischemic attack; VTE, venous thromboembolism.

**SUPPLEMENTAL FIGURE 2** Effectiveness and safety annualized clinical event rates in the first year (months 1–12) and second year (months 13–24; including repeated events^a,b^; sensitivity analysis).

^a^Excluding overall mortality and cardiovascular events as they cannot be repeated.

^b^Patients could contribute multiple events per year and contribute to both the first and second year annualized event rates with time under observation within each year used as the person-year denominator.

Numbers above the bars represent the clinical event counts (%/year).

CI, confidence interval; GI, gastrointestinal; ISTH, International Society on Thrombosis and Haemostasis.
